# Molecular Mechanisms of Fetal Tendon Regeneration Versus Adult Fibrous Repair

**DOI:** 10.3390/ijms22115619

**Published:** 2021-05-25

**Authors:** Iris Ribitsch, Andrea Bileck, Alexander D. Aldoshin, Maciej M. Kańduła, Rupert L. Mayer, Monika Egerbacher, Simone Gabner, Ulrike Auer, Sinan Gültekin, Johann Huber, David P. Kreil, Christopher Gerner, Florien Jenner

**Affiliations:** 1 VETERM, Equine Surgery Unit, Department of Companion Animals and Horses, University of Veterinary Medicine Vienna, 1210 Vienna, Austria; iris.ribitsch@vetmeduni.ac.at (I.R.); sinan.gueltekin@vetmeduni.ac.at (S.G.); 2 Department of Analytical Chemistry, Faculty of Chemistry, University of Vienna, 1090 Vienna, Austria; andrea.bileck@univie.ac.at (A.B.); Rupert.mayer@univie.ac.at (R.L.M.); 3 Chair of Bioinformatics, Department of Biotechnology, Boku University Vienna, 1180 Vienna, Austria; Alexander.aldoshin@boku.ac.at (A.D.A.); Maciej.kandula@boku.ac.at (M.M.K.); 4 Administrative Unit Veterinary Medicine, UMIT-Private University for Health Sciences, Medical Informatics and Technology GmbH, 6060 Hall in Tirol, Austria; monika.egerbacher@gmail.com; 5 Histology & Embryology, Department of Pathobiology, University of Veterinary Medicine Vienna, 1210 Vienna, Austria; simone.gabner@vetmeduni.ac.at; 6 Anaesthesiology and Perioperative Intensive Care Medicine Unit, Department of Companion Animals and Horses, University of Veterinary Medicine Vienna, 1210 Vienna, Austria; Ulrike.Auer@vetmeduni.ac.at; 7 Teaching and Research Farm Kremesberg, Clinical Unit for Herd Health Management in Ruminants, Department for Farm Animals and Veterinary Public Health, University of Veterinary Medicine Vienna, 1210 Vienna, Austria; Johann.Huber@vetmeduni.ac.at

**Keywords:** tendon healing, regeneration, inflammation, tendinopathy, proteomics, animal model, fetal

## Abstract

Tendinopathies are painful, disabling conditions that afflict 25% of the adult human population. Filling an unmet need for realistic large-animal models, we here present an ovine model of tendon injury for the comparative study of adult scarring repair and fetal regeneration. Complete regeneration of the fetal tendon within 28 days is demonstrated, while adult tendon defects remained macroscopically and histologically evident five months post-injury. In addition to a comprehensive histological assessment, proteome analyses of secretomes were performed. Confirming histological data, a specific and pronounced inflammation accompanied by activation of neutrophils in adult tendon defects was observed, corroborated by the significant up-regulation of pro-inflammatory factors, neutrophil attracting chemokines, the release of potentially tissue-damaging antimicrobial and extracellular matrix-degrading enzymes, and a response to oxidative stress. In contrast, secreted proteins of injured fetal tendons included proteins initiating the resolution of inflammation or promoting functional extracellular matrix production. These results demonstrate the power and relevance of our novel ovine fetal tendon regeneration model, which thus promises to accelerate research in the field. First insights from the model already support our molecular understanding of successful fetal tendon healing processes and may guide improved therapeutic strategies.

## 1. Introduction

Tendinopathy is a common, painful, and disabling musculoskeletal condition prevalent among athletes and sedentary subjects, afflicting 25% of the adult population as a result of trauma, overuse, or ageing [1,2]. In recreational and professional athletes, tendinopathies are amongst the most frequent musculoskeletal problems and account for 30–50% of all sports-related injuries [1,3,4]. Energy-storing tendons, such as the Achilles tendon, are most commonly affected [5]. Current treatment options are limited to pharmacological therapies, physiotherapy, and surgical intervention, which exclusively provide symptomatic relief and fail to restore the functional properties of injured tendons [6].

The onset and progression of tendinopathy is a multifactorial process affected by many intrinsic and extrinsic influences such as age, gender, genetics, anatomical variants, body weight, systemic disease, sporting activities, physical loading, occupation, and environmental conditions resulting in accumulated microdamage due to repetitive strain with weakening of the collagen cross-links, the non-collagenous matrix, and the vascular elements [1,7,8]. The pathogenesis of tendinopathy includes concurrent inflammatory, reparative and degenerative processes proceeding in parallel from early to late stages of the disease, causing clinical symptoms such as pain, focal tendon tenderness, and decreased strength and movement [7,9,10,11]. Unfortunately, the repair of adult tendons is slow and inefficient due to the low cellularity, vascularity, and metabolic rate of the tendon and fails to re-establish the physiologic three-dimensional tendon extracellular matrix (ECM) structure [12]. Thus, adult injured tendons fail to regenerate but form fibrous scar tissue with significantly inferior biomechanical properties that is prone to re-injury and chronic tendinopathy resulting in a significant impact on quality of life and high socioeconomic costs—the annual cost for tendon injury is estimated at USD 30 billion [2,3,13,14].

In contrast to the dysfunctional adult scarring repair, fetal tendons within the first two gestational trimesters heal regeneratively and achieve a restitutio ad integrum, including restoration of normal mechanical properties [15,16,17,18,19]. Recent studies have demonstrated that adult tenocytes retain an intrinsic capability to perform collagen fibrillogenesis similar to fetal tenocytes [20] and that mature cells exposed to a juvenile environment can rejuvenate [21], implying that the milieu, rather than intrinsic cellular function, inhibits regeneration of adult tendons and that fetal biochemical cues may have the potential to modulate adult tendon healing.

Thus, proteomic analyses of the age-specific responses of fetal and adult tendons to injury could reveal the molecular parameters dictating tendon regeneration, allowing the potential discovery of new therapeutic tools to improve adult healing. Although technically sophisticated, proteome profiling may offer a more in-depth understanding of disease etiopathogenesis as its interpretation is not affected by a possible disconnect between gene and protein expression levels [22].

**Figure 1:**
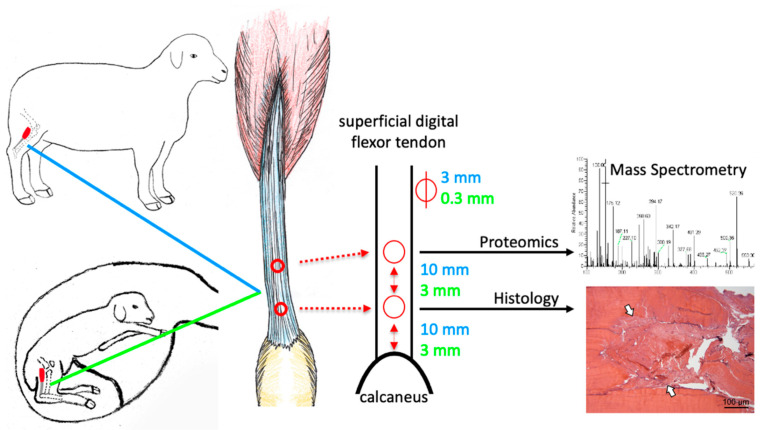
Tendon lesions were created in the superficial digital flexor tendon of adult and fetal (day 80 gestation) sheep using a biopsy punch of 3 mm (adult) and 0.3 mm (fetus) diameter in a caudocranial orientation. The tendon lesion locations and sizes are indicated in blue for adult and in green for fetal sheep. The distal lesion was created 10 mm (adult) and 3 mm (fetus) proximal to the calcaneus, the proximal lesion equidistant thereof. Samples were harvested at three days post-injury (pi), and the injury response relative to uninjured controls (for fetal sheep: uninjured twins) was compared between age groups using mass spectrometry and histology.

In the present study, we aim to (1) validate a standardized tendon lesion model facilitating comparison of tendon repair in adult and fetal sheep; (2) assess the feasibility, reproducibility, and biological relevance of fetal and adult tendon secretome proteomic analyses; and (3) compare fetal and adult protein regulation in response to acute tendon injury at day three post-injury (Figure 1).

## 2. Results

### 2.1. The Ovine Model Allows Studying of Fetal Tendon Regeneration

Ewes underwent laparotomy, uterotomy, and fetal manipulation with no postoperative complications or abortions. Fetal sheep at 80 days of gestation (gd, term = ∼150 days) had age-appropriate crown–anus lengths within the reported range of 10.1 ± 1.3 cm [23].

The landmarks for induction of superficial digital flexor tendon lesions were clearly identified in adult and fetal sheep and allowed placement of two lesions of 3 mm (adult) and 0.3 mm (fetal) diameter at a standardized distance to the calcaneus and each other (Figure 1).

### 2.2. Histological Long-Term Evaluation of Tendon Healing Confirmed Fetal Regenerative Versus Adult Scarring Tendon Repair

**Figure 2:**
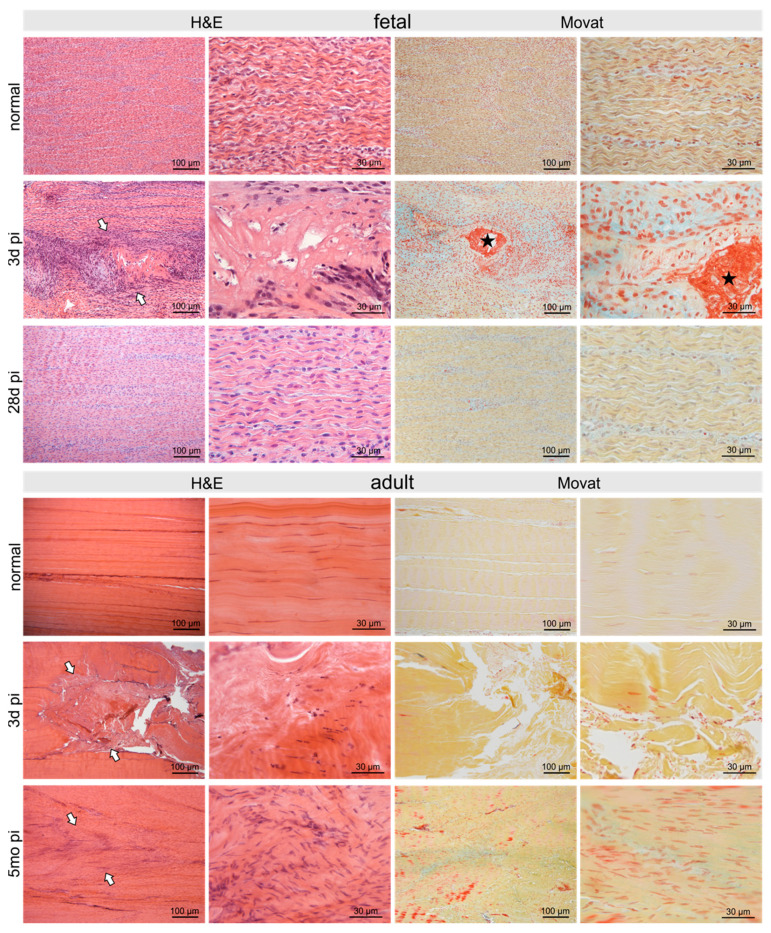
Micrographs show H&E- and Movat-stained normal control fetal and adult tendons and tendons after three days and 28 days/5 months post-injury (pi), respectively. The defect was clearly seen (arrows) after three days with comparable signs of damage in adult as well as fetal tendons: disruption of regular collagen fiber arrangement, acellular areas alternating with areas of hypercellularity. Fibrin deposition (asterisk) between damaged collagen fibrils was shown by Movat staining in fetal tendons only. Five months pi the damage was still clearly visible in the adult tendon (arrows) with hypercellular areas and irregular arrangement of collagen fibers as well as tenocytes. In contrast, in the fetal tendon, no signs of the previous injury were detectable after 28 days.

At three days pi adult and fetal tendons showed comparable signs of damage. In fetal sheep 28 days post-injury (pi), the tendon defect was no longer detectable histologically and no signs of previous injury remained (Figure 2). In contrast, in the adult tendon five months pi, the damage was still clearly visible with hypercellular areas and irregular arrangement of collagen fibers and tenocytes (Figure 2).

### 2.3. Injury- and Repair-Associated Histologic and Immunohistochemical Changes in Adult and Fetal Sheep Three Days Post-Injury

**Figure 3:**
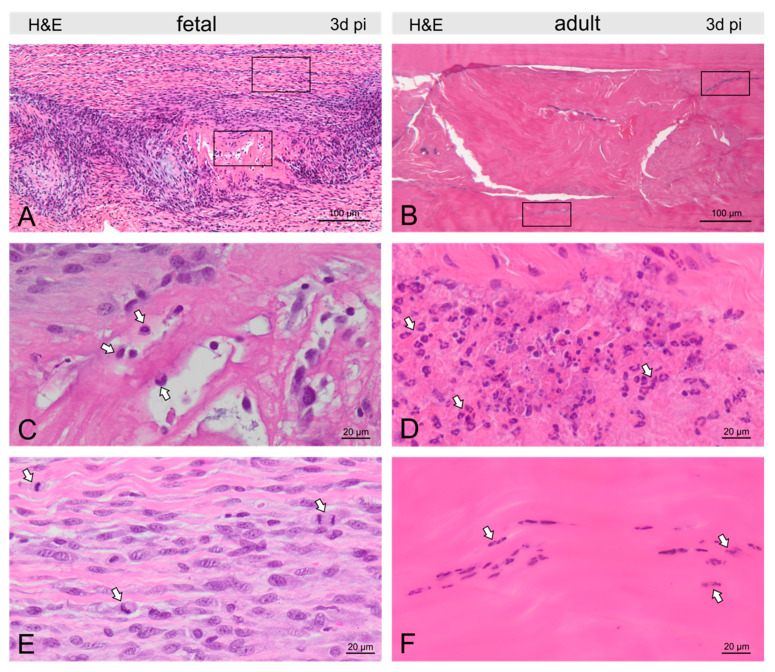
Micrographs show H&E-stained fetal (**A**) and adult (**B**) tendons three days post-injury (pi). Groups of macrophages (arrows) were found in and around the fetal tendon lesion (**C**), while in the adult tendon, nests of granulocytes (arrows) dominated the picture at the lesion site (**D**). In fetal tendons, we found few cells within but more cells around the lesion, also reflected by a remarkable number of mitotic figures (arrows, **E**). In adults, tenocytes were reduced in and around the lesion and condensation of chromatin was found in tenocytes (arrows) indicating cell death (**F**).

**Figure 4:**
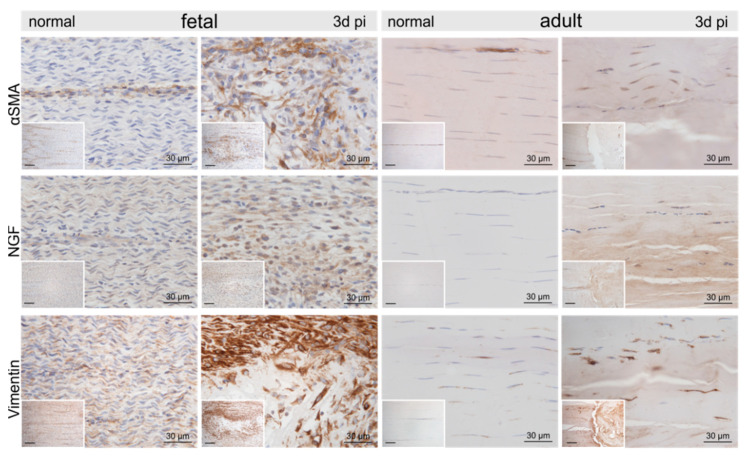
Representative micrographs of normal control and injured fetal and adult tendons three days post-injury (pi). Compared to the control tendons, we found an up-regulation of α-smooth muscle actin (αSMA), nerve growth factor (NGF), and vimentin within and around the lesion site in both age groups. Inserts show an overview at 10× magnification, scale bar = 100 µm.

**Figure 5:**
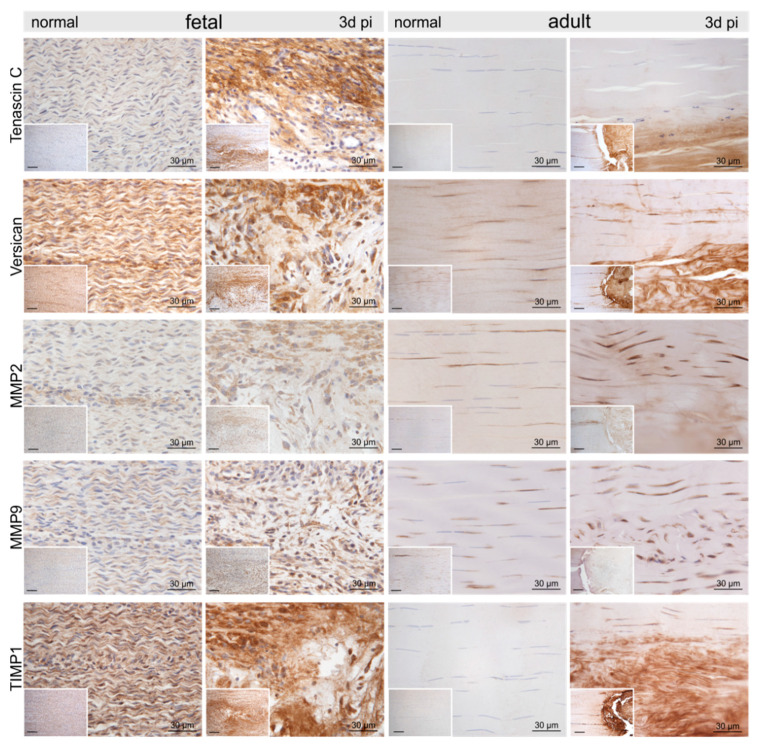
Representative micrographs of normal control and injured fetal and adult tendons three days post-injury (pi). Compared to the control tendons, we found an up-regulation of tenascin-C, versican, matrix metalloproteinase-2 (MMP2), MMP9, and tissue inhibitor of metalloproteinases-1 (TIMP1) within and around the lesion site in both age groups. Inserts show an overview at 10× magnification, scale bar = 100 µm.

In both fetal and adult tendons, the damage site was clearly visible at 3d pi, showing disrupted and disorientated collagen fibrils (Figure 2, Figure 3, Figure 4 and Figure 5). Extravascular erythrocytes and immune cells were found within the lesion in both age groups.

In fetal tendons, there was a trend for fewer cells within, but increased cellularity around, the lesion, corroborated by a remarkable number of mitotic figures (Figure 3). Lymphocytes and granulocytes were found rarely, but macrophages were prominent (Figure 3). In the lesion center, fibrin deposition between damaged collagen fibrils was seen in the Movat staining (Figure 2). In adults, tenocytes were reduced in the lesion and 300–400 μm circumference of the lesion and condensation of chromatin was found in the tenocytes indicating cell death (Figure 3). Nests of granulocytes were detected around the lesion, however, only a few lymphocytes and macrophages were found (Figure 3). Movat staining did not show any fibrin deposition.

Compared to the control tenocytes, cells strongly positive for α-smooth muscle actin (αSMA) exhibiting irregular shapes were identified in and around the fetal lesions. Strong staining for αSMA, nerve growth factor (NGF), and vimentin (VIM) was detected in tenocytes in the immediate vicinity (up to 300–400 μm distance) of the adult lesions (Figure 4). Higher tenascin-C (TNC), as well as versican (VCAN) immunoreactivity, was detected in cells and ECM within the lesion in both fetal and adult tendons compared to control samples (Figure 5). While matrix metalloproteinase-2 (MMP2) and MMP9 were detected in normal tenocytes of control tissue, a stronger immunopositivity was seen in cells within and around the lesion site in both age groups (Figure 5). Tissue inhibitor of metalloproteinases-1 (TIMP1) was not detectable in adult control tendons, whereas intense staining was found intracellularly and in the ECM in and around the lesions of both age groups (Figure 5).

### 2.4. Molecular Secretome Profiling by High-Resolution Mass Spectrometry

**Figure 6:**
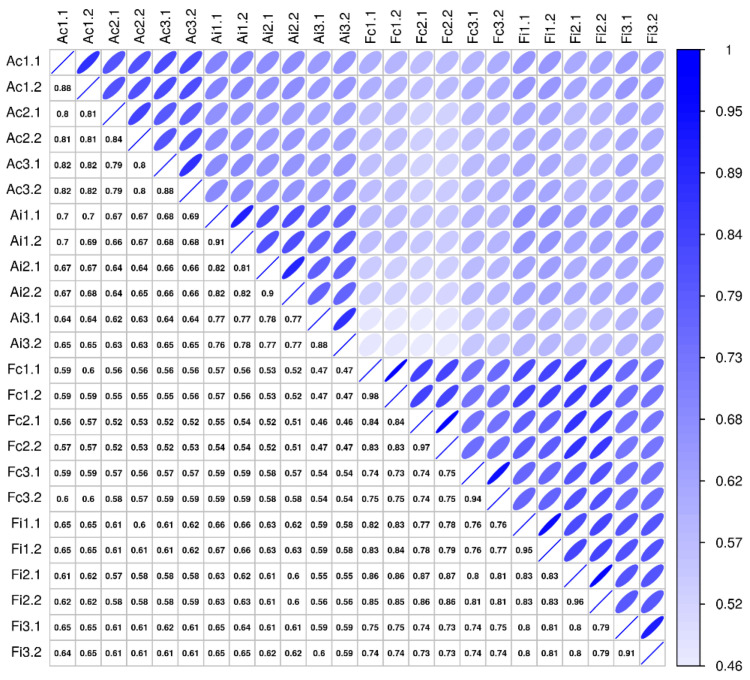
Sample correlation structure. This figure compares pairwise sample correlations: Spearman rank correlation coefficients are given in the boxes below the diagonal, and correlations are visualized above the diagonal (darker and narrower ellipses indicate higher correlations). Rows and columns show sample labels, where A/F = adult/fetal, c/i = control/injured, and #. # show biological and technical replicate numbers (*n* = 3 biological replicates per group, two technical replicates per biological replicate).

Comparative secretome analysis of control and injured (3 days postoperatively) tendon samples derived from adult and fetal sheep, using high-resolution mass spectrometry (MS), enabled the identification of 2489 distinct proteins (Supplementary Table S1). The technical measurement reproducibility was excellent, with variation clearly lower than the variation between biological replicates, indicating a high sensitivity of the proteomics profiling workflow (Figure 6). The general robustness of the analysis is reflected in the variance across biological replicates being small in relation to the examined biological effects, whether studying injury versus control or looking at differences between adult and fetal samples (Figure 6).

As described previously, tissue-derived secretome samples contain not only genuinely secreted proteins but also proteins originating from the interstitial fluid and cell debris, with some variance originating from the inherent complexity and heterogeneity of biological model systems and tissue samples [24,25]. Hence the proteins identified in the present work were classified accordingly, based on the UniProt database (www.uniprot.org (accessed on 4 March 2019), Supplementary Table S1). As expected, cytoplasmic proteins, derived from cell debris, represent the largest group of proteins (*n* = 1845), but also proteins originating from blood leaking from disrupted microvessels and interstitial fluid were abundant (*n* = 123) and showed characteristic features of injury.

**Figure 7:**
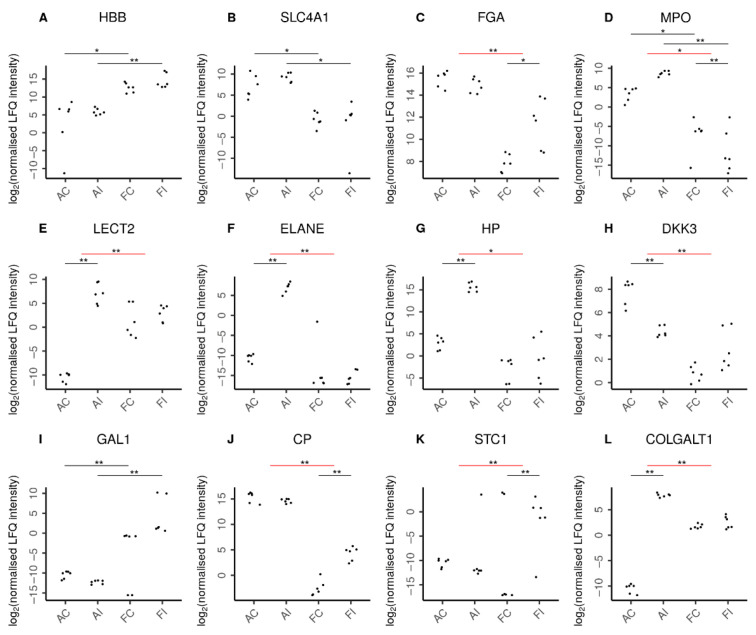
Selected proteins indicating characteristic features in adult and fetal animal samples. Log_2_ normalized LFQ intensities of each protein are shown for adult control (AC), adult injured (AI), fetal control (FC), and fetal injured (FI) tendon samples. The pairwise comparisons discussed are indicated by horizontal bars. Comparisons can be between two samples directly (black bars) or between the adult and fetal responses to injury (red bars). Statistical significance is then shown by double asterisks (**) and marginal statistical significance by a single asterisk (*). In line with expectations, (**A**) higher amounts of hemoglobin fetal subunit beta (HBB) were seen in fetal samples. Similarly, (**B**) higher levels of solute carrier family 4 member 1 (SLC4A1) reflected the increased presence of erythrocytes in adult samples. Remarkably, (**C**) fibrinogen chain alpha (FGA) was specifically increased only in fetal injury. Adults featured a higher presence of neutrophils (**D**) as reflected by myeloperoxidase (MPO) levels in these tissues. Moreover, the adult-specific response to injury was characterized by chemoattraction of neutrophils (**E**) as indicated by leukocyte cell-derived chemotaxin 2 (LECT2) and the secretion of antimicrobial proteins (**F**) by neutrophils such as neutrophil elastase (ELANE). In addition, the adult-specific response to injury featured up-regulation of pro-inflammatory proteins (**G**) such as the acute phase haptoglobin (HP) as well as the down-regulation of the anti-inflammatory (**H**) dickkopf WNT signaling pathway inhibitor-3 (DKK3). In contrast, fetal samples featured higher levels of anti-inflammatory proteins (**I**), such as galectin-1 (GAL1). Moreover, the fetal-specific response to injury featured up-regulation of anti-inflammatory proteins such as (**J**) the inflammation-resolving ceruloplasmin (CP) and (**K**) stanniocalcin-1 (STC1). The regulation of extracellular matrix proteins also played a more pronounced role in the adult response to injury with over a dozen ECM proteins specifically up-regulated in adults, as exemplified by (**L**) the collagen beta(1-O)galactosyltransferase 1 (COLGALT1). Supplementary Figure S1 shows further proteins characterizing the adult and fetal responses to injury.

Unsurprisingly, fetal hemoglobin (HBB) was detected at higher abundance levels in fetal samples (Figure 7), while adult hemoglobin subunit beta (HBBB) was predominantly found in adult samples. The presence of erythrocytes in adults was further confirmed by solute carrier family 4 member 1 (SLC4A1), which is expressed in the erythrocyte plasma membrane. In addition, we found erythrocyte-specific proteins increased upon injury only in adult but not fetal animals as suggested by the adult-specific significant increase of erythrocytic spectrin beta (SPTB) in response to injury. Interestingly, in contrast, fibrinogen chains alpha (FGA) and beta (FGB) were specifically increased only in fetal injury (Supplementary Table S2).

Functionally most relevant are the genuinely secreted proteins derived from local cells (Supplementary Table S1, *n* = 158). These highlighted a profound contribution of the innate immune system to the injury response, with a total of 74 proteins classified accordingly, including 17 antimicrobial proteins, 30 proteins involved in protease and peptidase enzymatic activities, and 13 pro-inflammatory proteins. Furthermore, eight proteins with chemokine activities, 24 proteins with growth factor and growth-factor-regulating activities, and 24 proteins described to affect cell differentiation were identified. Overall, inflammation was significantly up-regulated, specifically in adults following injury, while anti-inflammatory proteins were significantly up-regulated in the fetal response to injury (Table 1).

The strong induction of pro-inflammatory proteins characterizing the adult-specific response to injury (Figure 7, Supplementary Table S2) includes the adult-specific significant up-regulation of acute phase proteins serum amyloid A1 (SAA1) and haptoglobin (HP), as well as heme oxygenase 1 (HMOX1), a marker of oxidative stress. Also, chemokines indicative of chemoattraction and activity of neutrophils, including leukocyte cell-derived chemotaxin 2 (LECT2), CD177, neutrophil-expressed elastase (ELANE), antimicrobial neutrophil granular proteins lactotransferrin (LTF), and azurocidin 1 (AZU1), were specifically and significantly up-regulated in the adult response to tendon injury. The adult-specific pro-inflammatory response is further corroborated by the specific and significant up-regulation of several other inflammation-associated proteins known to promote fibrosis, such as S100 calcium binding protein A8 (S100A8), S100A9, S100A12, pentraxin-3 (PTX3), and integrin beta 1 (ITGB1).

In contrast, the response to injury in fetal animals was characterized by a fetal-specific up-regulation of anti-inflammatory proteins (Figure 7, Supplementary Table S2), including stanniocalcin-1 (STC1) and ceruloplasmin (CP). Conversely, we found proteins with anti-inflammatory activity such as the dickkopf WNT signaling pathway inhibitor-3 (DKK3) and the antimicrobial effector lysozyme significantly down-regulated specifically in the adult injury response. Also, annexin A4 (ANXA4) was significantly down-regulated in the adult injury response. Still, there was some anti-inflammatory effort in adult animals, as indicated by the specific up-regulation of the anti-inflammatory protein ArfGAP with SH3 domain, ankyrin repeat, and PH domain 1 (ASAP1/AMAP1), bringing its expression in adults to levels marginally exceeding those of fetal injured animals.

The regulation of ECM proteins also played a more pronounced role in the adult response to tendon injury (Supplementary Table S2). Thirteen ECM-associated proteins were specifically significantly up-regulated in adults, while prolyl 3-hydroxylase family member 4 (P3H4, LEPREL4), thrombospondin (THBS3), nidogen 1(NID1), peptidase D (PEPD) and procollagen C-proteinase enhancer protein (PCOLCE) were significantly down-regulated, specifically in adults. Among the ECM-associated proteins which were significantly up-regulated specifically in adult injury were proteases, such as matrix metalloproteinase 9 (MMP9), mannan-binding lectin serine peptidase-2 (MASP2), trefoil factor 2 (TFF2), and proteinase 3 (PRTN3), as well as tissue inhibitor of metalloproteinases 1 (TIMP1). Furthermore, cartilage-associated protein (CRTAP), procollagen-lysine,2-oxoglutarate 5-dioxygenase 3 (PLOD3), collagen type V alpha 1 chain (COL5A1), COL5A2, COL16A1, proteoglycan-4 PRG4, and collagen beta(1-O) galactosyltransferase 1 (COLGALT1) were also specifically and significantly up-regulated in adults (Figure 7L). In contrast, in the fetal-specific response to injury, only one ECM protein, proline, and arginine-rich end leucine-rich repeat protein PRELP, were significantly regulated, showing lower fetal abundances after injury. Stem cell marker galectin-1 (GAL1) was exclusively found in fetal samples (Figure 7I). Moreover, factors which are reportedly characteristic, albeit not exclusively, for stem cells, such as nidogen-1, connective tissue growth factor, secreted frizzled-related protein 2, and bone morphogenetic protein 1, were found particularly in fetal sheep post-injury potentially indicating stem cell activation in fetal compared to adult sheep in response to injury.

## 3. Discussion

Fetal mammals in the first two trimesters of gestation, in contrast to adults, display the ability for scar-free complete restoration of tissue and organ function following injury and thus constitute an ideal blueprint for regeneration. However, the underlying mechanisms and molecular pathways of this highly regulated process have not yet been elucidated. While differences in cellular density, proliferation rate, ECM composition, and synthetic function between fetal and adult individuals are evident, the strong correlation between an attenuated inflammatory response to injury and regeneration in the fetus, points to biochemical cues, including cellular inflammatory mediators, cytokines, enzymes, and growth factors, as critical elements for regeneration [26,27,28].

In this study, we confirmed the biological relevance and the technical feasibility of our novel ovine fetal tendon injury model and molecular profiling approaches. We demonstrated the complete regeneration of injured fetal tendons within 28 days, while adult injured tendons showed repair with scar tissue five months post-injury.

Sheep are a well-characterized and validated large animal model for regenerative approaches to tendinopathy [29], and the ontogeny of the ovine immune and inflammatory system and bone marrow niche has been very well described, establishing the sheep as an ideal model to elucidate fetal regeneration [19,30,31,32,33]. To be able to study the regenerative response in the presence of a fully functioning immune system and established ability to mount an inflammatory response, we chose to carry out this study in fetal sheep in the second trimester, at 80 days of gestation (gd, term = ∼150 days). Fetal sheep have a fully functioning immune system by 75 gd [34,35,36]. They produce leukocytes by 32 gd [37], tumor necrosis factor alpha (TNFα) and interleukin-1 (IL-1) as early as 30–40 gd [38], and obtain the capability to form significant amounts of specific antibodies in response to antigenic stimulation as early as 70 gd [39,40,41,42,43,44]. Fetal lambs reject orthotopic skin grafts and stem cell xenotransplants placed post 75–77 gd and mount an inflammatory response to injury by gestational day 65 [19,30,31,32,42,43,44]. Furthermore, large tendon wounds in fetal lambs have been shown to be associated with inflammatory cell infiltration and up-regulation of the pro-inflammatory mediators IL-6 and IL-8 [19], thus demonstrating that this model allows regenerative healing to be examined without the confounding variable of an undeveloped immune system.

While the adult injury response is well characterized and can be divided into an inflammatory phase of 3–5 days, a proliferative phase of 3–6 weeks, and a remodeling phase of up to one year duration, the timeline of the fetal injury response is not yet established. However, up-regulation of pro-inflammatory genes has been shown in fetal tendons three days following injury [19]. Therefore, as inflammation is one of the key injury responses hypothesized to crucially contribute to the difference between adult and fetal healing, tissues were harvested and analyzed three days after injury, within the established time window of inflammation for both adult and fetal individuals. The inflammatory response to injury is highly conserved in all tissues and plays important roles in normal and pathological healing [45,46]. Following injury, resident mast cells may degranulate, releasing pro-inflammatory mediators, which initiate the inflammatory cascade and attract first neutrophils and then macrophages and mast cells from nearby tissues and from the circulation to the wound site [46,47].

In this study, at three days post-injury, fetal tendon wounds were associated with very few granulocytes but prominent recruitment of macrophages, while in contrast, adult tendon wounds were accompanied by only a few macrophages but nests of granulocytes, mostly neutrophils, and a rim of decaying tenocytes around the lesion.

These histological assessments were corroborated by the secretome analysis data obtained by exploratory whole-proteome profiling, supporting mutual and independent validation as outlined in the following. Secretome profiling may suffer from characteristic problems associated with the analysis of bulk fractions, representing a mixture of different contributors. Some observations may thus result from experimental conditions and be considered less relevant, as, e.g., the apparently higher occurrence of erythrocyte components in adult samples most plausibly caused by the larger vessel size compared to the fetus. However, 27 proteins characteristic for neutrophils were found more up-regulated upon injury in adult than in fetal animals with marginal significance (Table 1), supporting histological assessment. In addition, chemokines known to attract neutrophils were found specifically up-regulated in the adult model upon injury. The tissue response to the potentially damaging effects of neutrophil activities, which may contribute to adult patho-mechanisms, was evidenced by the strong induction of HMOX1, a marker molecule for oxidative stress, as well as the specific up-regulation of several proteases known to degrade extracellular matrix such as MMP9, mannan-binding lectin serine peptidase-2 (MASP2), proteinase-3 (PRTN3), and trefoil factor-2 (TFF2) [48]. The marked up-regulation of various ECM proteins, including COLGALT1, PRG4, COL5A1, and COL5A2, may point to the active attempts of the stromal cells to regenerate the injured tendon tissue. However, histologic assessment showed an irregular ECM structure in the adult, indicating disturbed coordination of ECM deposition, maturation, and functional fibril formation.

Remarkably, COL5 has been described to potentially form autoantigens in the adult, resulting in systemic sclerosis and fibrosis [49]. Furthermore, COL5, while vital in regulating fibrillogenesis, is associated with smaller fibril diameters.

Also, histologic observations regarding the increased occurrence of macrophages in fetal tendons were verified by proteome profiling. While 13 proteins promoting inflammation characteristic for innate immune responses were found up-regulated in adults upon injury, these proteins were considerably less affected in fetal samples.

As much lower chemokine increase was observed in fetal conditions upon injury, and chemokines attract neutrophils via the formation of concentration gradients [50], the secretome analyses results indeed indicate recruitment of neutrophils to the site of injury mainly in the adult animals. In contrast, in fetal samples, anti-inflammatory proteins, several inflammation-resolving molecules, anti-oxidative proteins, and stem cell markers were found up-regulated upon injury. These data point to a relevant activity of fetal stem cells synergistically supported by systemic factors contributed by the liver, such as CP, and local factors derived from stromal cells upon fetal healing. It is hence reasonable to assume that the attenuation of inflammation and inflammation-related tissue damage is highly relevant for fetal tendon healing.

The molecular profiling data thus suggest that the innate immune system may cause substantial tissue damage in adults, based on a kind of molecular misunderstanding, which has also been described for inflammation-induced tumorigenesis [51,52,53]. The defense against potential pathogens appears to be evolutionarily prioritized over regenerative processes in the adult. However, understanding the signaling cascade from the initial dissemination of alarmins and chemokines to the recruitment and activation of innate immune cells causing undesired collateral damage may identify powerful therapeutic targets. At this point it is important to note, that for animal welfare purposes, adult sheep were allowed free movement following surgery. While fetal sheep move in the uterus subjecting their tendons to some strain, they do not bear weight. Hence adult tendons were exposed to more strain post-injury, which may have affected the inflammatory response. While early mechanical loading stimulates and improves tendon healing, the effects are dose-dependent and strong loading has been shown to increase the expression of genes involved in the inflammatory response [54,55,56,57,58]. While we acknowledge this limitation of our study, it does reflect the circumstances of natural tendon healing and allows interpretation in this context.

Rather unexpectedly, most cytokines and chemokines were also found expressed at high levels in fetal control samples. However, immune responses associated with high levels of cytokines and chemokines in the fetus have been described to be necessary to promote healthy pregnancy [59], explaining the baseline expression.

Hence, the present data clearly suggest that the inhibition of inflammatory cell damage supported by the accelerated clearance of debris and activation of stem cells may be crucial for the successful healing processes observed in fetal animals.

In summary, the present findings reveal the biological significance of the novel ovine fetal tendon regeneration model and of the analytical approach, and qualitatively validate the model as a baseline for the scientific community for future quantitative studies. The data contribute to our molecular understanding of successful tendon regeneration and point to a concerted effort to limit innate immunity-induced cell damage in the fetal environment, emphasizing the importance of immunomodulation for regeneration. The results encourage further studies to examine the fundamental mechanisms of fetal regenerative tendon repair, with the goal to identify therapeutic targets to modulate the response of the organism to injury to achieve regenerative tenogenesis in adults.

## 4. Materials and Methods

### 4.1. Animal Model

We created standardized tendon lesions in musculoskeletally mature, adult (2–5 years, body weight 95 ± 12 kg), healthy, female, non-gravid merino sheep (*Ovis aries*) and fetal lambs (gestational day 80, term = approximately 145 days) with approval of the national (Commission for Animal Research of the Austrian Federal Ministry of Science, Research and Economy) and institutional (Ethics and Animal Welfare Committee of the University of Veterinary Medicine, Vienna) animal welfare committees (approval numbers 68.205/0155-WF-/V/3b/2014 and 68.205/0028-II/3b/2014).

Adult sheep were treated with antibiotics (ceftiofur, 2.2 mg/kg bodyweight i.m. once daily) peri-operatively. Pain management was provided with morphine (morphine-hydrochloride-trihydrate, 0.1 mg/kg bodyweight i.m. 2–6 times daily) to avoid anti-inflammatory drugs, which would influence the result of the study. Adult sheep were placed under general anesthesia using premedication with midazolam (0.2–0.4 mg/kg i.v.) and butorphanol (0.1 mg/kg i.v), anesthesia was induced with ketamine (3–5 mg/kg i.v.) and maintained with 1–2% isoflurane in 100% oxygen combined with remifentanil continuous rate infusion (0.5 µg/kg/min).

For the fetal subjects, only twin pregnancies were included to provide a twin lamb as an uninjured source of matched tissues on a background of low genetic variation to allow differentiation between protein secretion of regular fetal development and fetal response to tendon injury. Following a standard ventral-midline laparotomy and uterotomy, one twin was randomly assigned to the injured group, and its twin was used as uninjured age-matched control.

For both adult and fetal sheep, the superficial digital flexor tendon was exposed immediately proximal to the calcaneus via a medial approach and two 3 mm (adult) and 0.3 mm (fetal) diameter (approximately 1/3 of the tendon width for both adult and fetal sheep) full-thickness tendon lesions were created in a caudocranial direction in both hindlimbs (Figure 1). The incision over the tendon was closed in one dermal layer using 6-0 monofilament nylon (Monosof, Covidien, Minneapolis, MN, USA) in fetal lambs and in two layers using 2-0 monofilament absorbable polyester (Biosyn, Covidien) for the subcutaneous tissue and 2-0 monofilament nylon for the skin in adult sheep. Adult animals were allowed full weight-bearing immediately after surgery. Fetal lambs were returned to the uterus, and the uterotomy and the laparotomy incision were closed routinely (uterotomy and subcutaneous tissues: 2-0 monofilament absorbable polyester (Biosyn, Covidien), linea alba: one braided absorbable glycolide/lactide copolymer (Polysorb, Covidien), skin: 2-0 monofilament non-absorbable nylon (Monosof, Covidien)).

### 4.2. Tissue Harvest

Adult sheep (*n* = 3/group) were euthanized at day 0 (uninjured control), day 3 and 5 months (adult) post-injury and fetal twin sheep (*n* = 3/group) were euthanized following retrieval via a routine caesarean section at days 3 and 28 days after surgical tendon lesion induction. The superficial digital flexor tendons were excised, and perilesional tendon tissue (3 mm from the lesion in adults and 1 mm from the lesion in fetal sheep) was harvested (Figure 1). Left and right tendon samples were randomly assigned to mass spectrometry (only day 3 and control samples) and histology.

### 4.3. Histology and Immunohistochemistry

For histological analysis, tendons were fixed in 4% buffered formalin. After embedding in paraffin, 4 μm thick sections were cut and mounted on 3-aminopropyltriethoxysilane (APES)-glutaraldehyde-coated slides (Sigma-Aldrich, Vienna, Austria). Consecutive sections were stained with hematoxylin and eosin (H&E) and Movat pentachrome stain (Morphisto, Offenbach am Main, Germany). For immunohistochemistry, sections were deparaffinized, rehydrated, and endogenous peroxidase was blocked with 0.6% hydrogen peroxide in methanol (15 min at room temperature). Nonspecific binding of antibodies was prevented by incubation with 1.5% normal goat serum (Dako Cytomation, Glostrup, Denmark) in phosphate-buffered saline (PBS; 30 min at room temperature). Primary antibodies (αSMA, NGF, TNC, VCAN, MMP2, MMP9, TIMP1, and VIM; Supplementary Table S4) were incubated overnight at 4 °C. An appropriate BrightVision Peroxidase system (Immunologic, Duiven, The Netherlands) was used, and peroxidase activities were localized with diaminobenzidine (DAB Quanto Chromogen TA-125-QHDX, Thermo Fisher Scientific, Waltham, MA, USA). Cell nuclei were counterstained with Mayer’s hematoxylin. Tissue from adult sheep mammary glands and sections of the viscerocranial part of the fetal sheep head served as positive controls. For negative controls, the primary antibody was omitted.

### 4.4. Secretome Analysis by Mass Spectrometry

Tendon samples were incubated in serum-free RPMI (Gibco, Life Technologies, Vienna, Austria) supplemented with 100 U/mL penicillin and 100 µg/mL streptomycin (ATCC, LGC Standards GmbH, Wesel, Germany) for 6 h under standard conditions (37 °C and 5% CO_2_). Afterwards, the medium was sterile-filtered through a 0.2 µm filter and precipitated overnight with ice-cold ethanol at −20 °C. After precipitation, proteins were dissolved in sample buffer (7.5 M urea, 1.5 M thiourea, 4% CHAPS, 0.05% SDS, 100 mM dithiothreitol (DDT)) and protein concentrations were determined using Bradford assay (Bio-Rad Laboratories, Munich, Germany).

Twenty micrograms of each protein sample were used for filter-aided digestion as described previously [60,61,62,63,64]. Briefly, 3 kD molecular weight cut-off filters (Pall Austria Filter GmbH, Vienna, Austria) were conditioned with MS grade water (Millipore GmbH, Vienna, Austria). Protein samples were concentrated on the pre-washed filter by centrifugation at 15,000× *g* for 15 min. After reduction with DTT (5 mg/mL dissolved in 8 M guanidinium hydrochloride in 50 mM ammonium bicarbonate buffer (ABC buffer), pH 8) and alkylation with iodoacetamide (10 mg/mL in 8 M guanidinium hydrochloride in 50 mM ABC buffer), samples were washed, and 1 µg trypsin was added prior to incubation at 37 °C for 18 h. After enzymatic digestion, peptide samples were cleaned with C-18 spin columns (Pierce, Thermo Scientific, Vienna, Austria), dried and stored at −20 °C until analysis.

For mass spectrometric analyses, dried samples were reconstituted in 5 µL 30% formic acid (FA) containing 10 fmol of four synthetic standard peptides each and diluted with 40 µL mobile phase A (H_2_O:ACN:FA = 98:2:0.1). Ten microliters of the peptide solution were loaded onto a 2 cm × 75 µm C18 Pepmap100 pre-column (Thermo Fisher Scientific, Vienna, Austria) at a flow rate of 10 µL/min using mobile phase A. Afterwards, peptides were eluted from the pre-column to a 50 cm × 75 µm Pepmap100 analytical column (Thermo Fisher Scientific, Vienna, Austria) at a flow rate of 300 nL/min and separation was achieved using a gradient of 8% to 40% mobile phase B (ACN:H_2_O:FA = 80:20:0.1) over 95 min. For mass spectrometric analyses, MS scans were performed in the range of *m/z* 400–1400 at a resolution of 70,000 (at *m/z* = 200). MS/MS scans of the eight most abundant ions were achieved through HCD fragmentation at 30% normalized collision energy and analyzed in the orbitrap at a resolution of 17,500 (at *m/z* = 200). All samples were analyzed in duplicate.

### 4.5. Data Analysis and Statistics of Mass Spectrometry Experiments

Protein identification as well as label-free quantitative (LFQ) data analysis was performed using the open-source software MaxQuant 1.3.0.5, including the Andromeda search engine [65]. Protein identification was achieved by searching against *Ovis aries* in the UniProt Database (version 09/2014 with 26,864 entries), allowing a mass tolerance of 5 ppm for MS spectra and 20 ppm for MS/MS spectra, as well as a maximum of two missed cleavages. In addition, carbamidomethylation on cysteines was included as fixed modification, whereas methionine oxidation, as well as *N*-terminal protein acetylation, were included as variable modifications. Furthermore, search criteria included a minimum of two peptide identifications per protein, at least one of them unique, and the FDR calculation based on *q*-values performed for both peptide identification as well as protein identification, less than 0.01. Prior to statistical analyses, proteins were filtered for reversed sequences, contaminants, and a minimum of three independent identifications per protein.

Missing values were replaced by a global *ε*, set to the minimum intensity observed in the entire data set divided by 4. This sensitivity based pseudo-count reflects the prior belief of non-observed protein expression, maintaining a lower bound of a 4-fold change for differences to proteins not observed in one sample group, thus maintaining sensitivity while improving specificity by mitigating the effects of random non-observations. The Spearman rank correlations between samples are not affected by this transform. For the visualization of the sample correlation structure in that figure (Figure 6), ellipses were plotted as (*x*, *y*) = (cos(*θ* + *d*/2), cos(*θ*-*d*/2)), where *θ* ϵ [0,2π) and cos(*d*) = *ρ*, with *ρ* the Spearman rank correlation coefficient [66].

MaxQuant (Version 1.6.8.0), including the in-built Andromeda search engine, was used for label-free quantification [65]. For identification, we used only non-redundant Swissprot entries with at least two peptides assigned per protein (of which one needed to be unique). The first and main search peptide tolerance were 50 and 25 ppm, respectively. The false discovery rate (FDR) was fixed to 0.01 on the peptide and protein level. The statistical evaluation was performed with Perseus software (Version 1.6.6.0) using LFQ intensities of the MaxQuant result file [67]. After filtering potential contaminants, the LFQ values were log_2_-transformed. The final data set was further investigated using web-based applications based on gene ontology annotations referring to human orthologues (e.g., DAVID Bioinformatics Resources 6.8).

The statistical analysis to assess the significance of single-protein responses to injury was done using empirical Bayes regularized tests for linear contrasts as implemented in the R package limma [68], following the methods proposed by Smyth [69]. The raw *p* values were adjusted by the Benjamini and Yekutieli method for strong control of the false discovery rate (FDR) [70]. Seeing that standard independent enrichment tests also yielded biologically uninformative hits for random protein lists, indicating clear system-level shifts, instead, a competitive enrichment test was employed for proteins associated with processes of interest, as implemented by the cameraPR function of limma [71]. The curated protein groups of interest were compiled manually (Supplementary Table S3). The test yields a direction of change (‘up’ or ‘down’) and an FDR value. We report results for all groups tested, so the individual FDR values apply to the respective tests. Unless stated otherwise, for all tests, the commonly used threshold of *q* < 0.05 was applied for calling statistical significance, and a threshold of *q* < 0.1 was used for marginal significance.

## Figures and Tables

**Table 1:** Summary table for competitive group enrichment tests showing results for pairwise comparisons between the adult and fetal groups. The adult (a) injury response comparing adult injured versus adult control samples, the fetal (f) injury response comparing fetal injured versus fetal control samples, and the difference between the injury responses (fvsa) comparing (f) versus (a) are detailed in the table. The direction of the change is indicated with a plus symbol for up-regulation and a minus symbol for down-regulation. We use the common FDR threshold of *q* < 0.05 for statistical significance. Supplementary Table S3 lists the proteins tested for each of the groups.

Group	a.*q*-Value	a.dir	f.*q*-Value	f.dir	fvsa.*q*-Value	fvsa.dir
Antimicrobial	5.25 × 10^−5^	+	2.46 × 10^−3^	+	5.97 × 10^−2^	-
Pro-inflammatory	3.68 × 10^−6^	+	1.31 × 10^−2^	+	3.90 × 10^−3^	-
Anti-inflammatory	4.61 × 10^−1^	-	1.60 × 10^−2^	+	9.71 × 10^−2^	+
Redox-regulating proteins	2.27 × 10^−1^	-	8.24 × 10^−1^	+	1.87 × 10^−1^	+
Protease/peptidase and regulators	5.29 × 10^−1^	+	9.39 × 10^−1^	+	7.89 × 10^−1^	-
ECM-related	3.80 × 10^−1^	+	9.91 × 10^−1^	+	7.98 × 10^−1^	-
Platelet-related	4.85 × 10^−1^	+	1.95 × 10^−1^	+	9.97 × 10^−1^	+
Acute phase-response	9.94 × 10^−1^	+	6.06 × 10^−3^	+	3.89 × 10^−1^	+
Erythrocyte-related	7.42 × 10^−1^	+	9.12 × 10^−1^	-	6.57 × 10^−1^	-

## Data Availability

The datasets generated and analyzed during the current study are available from the corresponding authors on reasonable request.

## Supplementary Materials

The following are available online at https://www.mdpi.com/article/10.3390/ijms22115619/s1.
