# Supplementary material for: Molecular Mechanisms of Fetal Tendon Regeneration Versus Adult Fibrous Repair
Source: Int J Mol Sci. 2021 May 25;22(11):5619. doi: 10.3390/ijms22115619 (PMC8198517; doi:10.3390/ijms22115619)
Supplement: Supplementary file 1 [file ijms-22-05619-s001.zip › Figure S1.pdf]

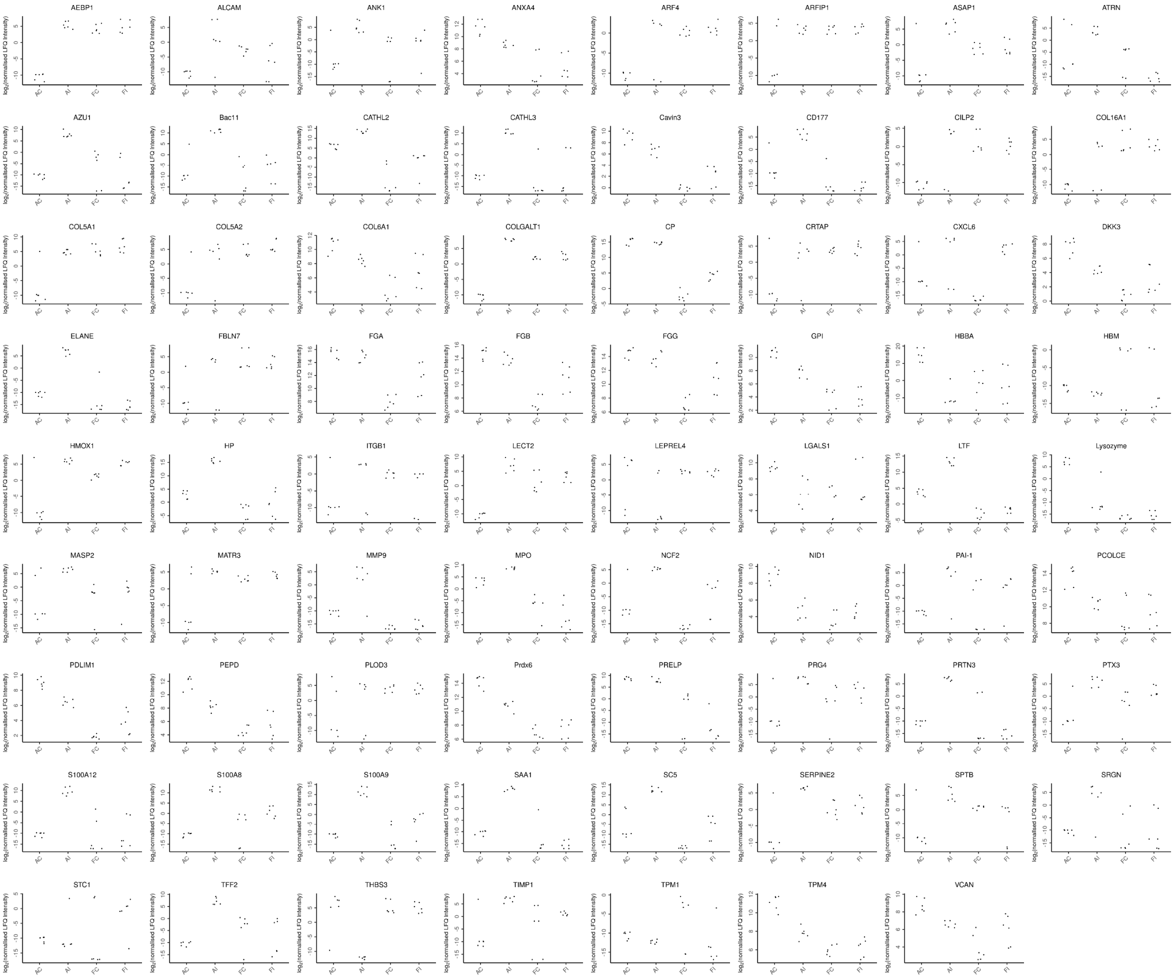

Figure S1. Proteins characterizing the adult and fetal responses to injury. Log_2 normalized LFQ intensities are shown for each protein for adult control (AC), adult injured (AI), fetal control (FC), and fetal injured (FI) tendon samples. Details of the statistical significance of changes in pairwise comparisons are listed in Supplementary Table S2.
