# Supplementary material for: Molecular Mechanisms of Fetal Tendon Regeneration Versus Adult Fibrous Repair
Source: Int J Mol Sci. 2021 May 25;22(11):5619. doi: 10.3390/ijms22115619 (PMC8198517; doi:10.3390/ijms22115619)
Supplement: Supplementary file 1 [file ijms-22-05619-s001.zip › Table S4.pdf]

Table S4. Sources, pre-treatments, and dilutions of the antibodies used for histology.

Antibody	Clone	Dilution	Pre-treatment	Source
αSMA	1A4, mouse	1:400	No pre-treatment	Dako, Glostrup, Denmark
NGF	E-12, mouse	1:200	0.01 M Citrate buffer pH 6 (2 h at 65 °C)	Santa Cruz Biotech., CA, USA
Vimentin	V9, mouse	1:500	0.01 M Citrate buffer pH 6 (2 h at 65 °C)	Dako, Glostrup, Denmark
Tenascin C	polyclonal, rabbit	1:200	0.01 M Citrate buffer pH 6 (2 h at 65 °C)	Santa Cruz Biotech., CA, USA
Versican	polyclonal, rabbit	1:6000	1 mg/mL Collagenase * (10 min at RT)	Abcam Cambridge, UK
MMP2	polyclonal, rabbit	1:400	0.01 M Citrate buffer pH 6 (2 h at 65 °C)	Abcam Cambridge, UK
MMP9	polyclonal, rabbit	1:400	0.01 M Citrate buffer pH 6 (2 h at 65 °C)	Abnova, Taipeh, Taiwan
TIMP1	polyclonal, rabbit	1:300	0.01 M Citrate buffer pH 6 (2 h at 65 °C)	NeoMarkers, CA, USA
